# Dynamic viscosity recovery of electrospinning solution for stabilizing elongated ultrafine polymer nanofiber by TEMPO-CNF

**DOI:** 10.1038/s41598-020-69136-2

**Published:** 2020-08-10

**Authors:** Shougo Higashi, Takayuki Hirai, Masato Matsubara, Hiroaki Yoshida, Atsushi Beniya

**Affiliations:** Toyota Central R&D Laboratories, Inc, 41-1, Nagakute, Aichi 480-1192 Japan

**Keywords:** Nanoscale materials, Nanowires

## Abstract

Electrospinning is a widely used production method for nanoscale fine polymer fiber fabrics. An ultrafine fiber made of polymers such as polyvinylpyrrolidone (PVP) polyacrylic acid (PAA) has immense potential for applications in air filters, batteries, and biosensors. However, producing fabrics with long uniformly distributed ultrafine fibers of a mean diameter below ~ 200 nm is still a challenge, because such elongated-ultrafine fibers tend to break into beads before they reach the collector. Here, we exploits the thixotropy of the solution given by the addition of 2,2,6,6-tetramethylpiperidin-1-oxyl-oxidized cellulose nanofibers to recover the solution viscosity for stabilizing the electrostatically elongated nanofibers, whereby the solution is smooth in the syringe needle owing to the shear force but regain its original viscosity after being freed from electrostatic force. Using this method, we successfully fabricated a non-woven ultrafine-long nanofiber made of PVP and PAA with a mean diameter as low as ~ 90 nm with a negligible number of beads.

## Introduction

**Figure 1:**
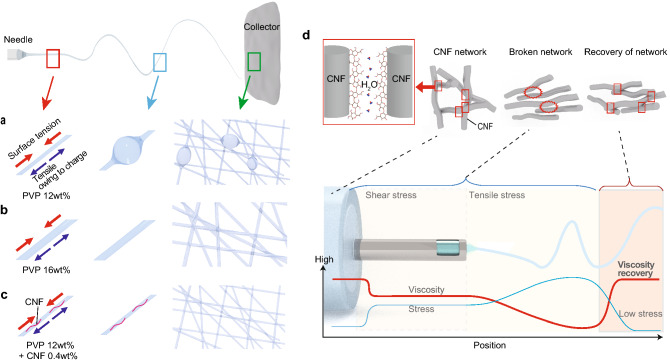
Concept for achieving a non-woven textile consisting of ultrafine fibers without beads. (**a**) A low-polymer-concentration solution produces fine fibers below 100 nm but forms a beady structure because of its low viscosity. (**b**) The high viscosity of the high-polymer-concentration solution overcomes the surface tension; however, the solution produces thick fibers over 100 nm. (**c**) By adding water-soluble TEMPO-oxidized cellulose, the reinforced solution overcomes the surface tension and achieves the desired fiber structure. (**d**) Schematic images of the DVT method for stabilizing the most elongated state of the formed nanofiber to prevent breakage into beads before it reaches the winder based on the thixotropic property of the solution introduced by CNF, showing hypothetical curves of the shear stress and viscosity during electrospinning at each position.

Non-woven textiles made of nanometer-scale polymer fibers play important roles in industry^1^. Various kinds of polymers dissolved in both non-aqueous and aqueous solutions, which usually contain ~ 4–20 wt.% of the polymer, have been successfully electrospun for different applications, such as filtration^2^, protective clothing^3^, battery separators^4^, nanocatalyst scaffolds^5^, tissue engineering^6,7,8^, and drug delivery^9,10^. Electrospinning is the most widely studied method for producing nanofiber textiles, and efforts to improve this process are ongoing^11^. In general, for needle electrospinning, the primary parameters affecting the final structure of the electrospun non-woven textile are the viscosity, dielectric constant, surface tension of the solution, solution feed rate, and bias voltage applied to the polymer solution between the syringe and collector, all of which determine the constituting fiber diameter and morphology^11,12^. These parameters are essential even for the state-of-the-art electrospinning method, and conditions for fabricating textiles with uniform fine polymer fibers have been widely investigated. In particular, electrospun fiber textiles made of polyvinylpyrrolidone (PVP), which is a representative water-soluble, non-toxic, biocompatible polymer, have been widely studied, particularly the morphology of the electrospun fibers^13^. As with other polymers, the viscosity and fiber diameter decrease with decreasing concentration of PVP in the electrospinning solution^13^. For low-concentration PVP solutions, ultrafine fibers of diameter ~ 20–50 nm have been observed locally^14^. However, when the polymer concentration is significantly low, beady structures start to form in the middle of the fibers for fiber diameters typically below ~ 200 nm^15,16^, because of the surface tension minimizing the total surface free energy^17^. Micron-sized beads appear as the PVP concentration further decreases because of its low viscosity, which cannot overcome the surface tension of the solution, as illustrated in Fig. 1a.

To overcome the surface tension, a high PVP concentration, and hence, high viscosity, are required; however, these conditions result in a thick fiber (Fig. 1b). Note that this scenario is not specific to the PVP system; in general, electrospinning non-woven textiles consisting of fibers with an average diameter below ~ 100 nm results in morphology with a beady shape^18^.

To suppress the formation of beads, a salt is often introduced into the polymer solution to add more charge (or to increase the conductivity) to overcome the surface tension^19^. Small-diameter fibers with short lengths mixed with thick fibers have successfully been fabricated^20^, however, forming long, uniform nanofibers without a large number of beads or thick fibers remains challenging with many polymers. Thus, a novel concept and method that do not merely improve the conductivity of the solution to elongate the fiber, but stabilize the fiber in this elongated state, are highly sought after to prevent breakage into beads before the fiber reaches the winder or fiber collector.

Thixotropy is a unique time-dependent property. It has recently been exploited to enable ice cream to maintain its shape, preventing deformation due to melting until it receives some stress^21^. 2,2,6,6-tetramethylpiperidin-1-oxyl (commonly known as TEMPO)-oxidized cellulose nanofibers (CNF) added aqueous solution is well-known to have this property^22,23^. With this in mind, we conceived the idea of adding cellulose into a low-concentration PVP solution to alter the electrospinning solution thixotropic, in order to stabilize the ultrafine nanofibers elongated during electrospinning and prevent the fragmentation of the fiber into beads. Adding TEMPO-CNF not only increases the solution viscosity but also can dynamically change this viscosity such that the solution inside the syringe needle is smooth and suitable for electrospinning owing to the shear stress; however, the solution viscosity is recovered when discharged from the edge of the syringe needle and after being started freed from the large tensile stress owing to the bias voltage applied during electrospinning. This increase in viscosity prevents deformation of the electrostatically elongated ultrafine fibers into beads. The concept of this dynamic viscosity tuning (DVT) method is summarized in Fig. 1c,d. To the best of our knowledge, this is the first experimental study to directly exploit the thixotropic properties of a CNF solution in electrospinning.

In this study, we fabricate a non-woven ultrafine nanofiber textile of PVP with an average fiber diameter of ~ 90 nm and a negligible number of beads using DVT. Three parameters are considered in DVT, namely, the TEMPO-CNF concentration, solution feed rate, and syringe needle diameter; here, the latter two were fixed. To demonstrate the DVT concept, we first investigate PVP aqueous solution concentrations that resulted in ultrafine fibers with an average diameter of approximately 100 nm with beads; then, we add TEMPO-CNF into the PVP aqueous solution to obtain a bead free textile. Solution conductivity is one of the important parameters affecting the fiber diameter and morphology. We also investigate the effect of solution conductivities of prepared solutions and discuss how they affect the resulting fiber diameter and morphology.

## Results

### PVP aqueous solution without TEMPO-CNF

**Figure 2:**
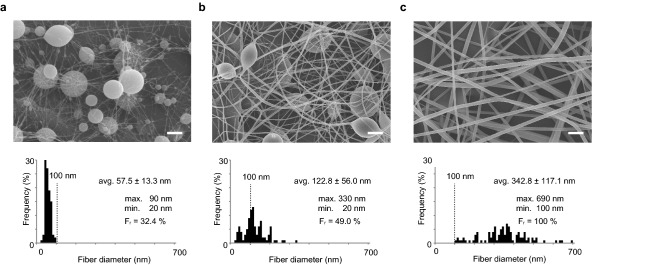
SEM observations of electrospun PVP. Electrospun fibers with (**a**) 8 wt.%, (**b**) 12 wt.%, and (**c**)16 wt.% PVP aqueous solutions. Finer fibers are formed as the PVP concentration decreases; however, the number of beads increases. Scale bars, 2 μm (**a**–**c**). Inset values are the average ± standard deviation. F_r_: fiber ratio.

First, we prepared aqueous PVP solutions of three different concentrations: 8, 12, and 16 wt.% of PVP with MilliQ water (18.2 MΩ). Figure 2a shows typical scanning electron microscopy (SEM) images of electrospun fibers prepared with a low concentration of 8 wt.% of PVP aqueous solution. The average fiber diameter was ~ 60 nm, and it exhibited a large number of micron-sized beads, which is consistent with previous research^15^. The fiber ratio (F_r_), which represents the quality of the textile and will be defined below, was estimated by analyzing the obtained SEM image (Supplementary Note 1, Supplementary Figs. 1–3). The number of beads decreased for the 12 wt.% PVP aqueous solution, and we still observed the ultrafine nanofibers (Fig. 2b). The beads disappeared for the most concentrated viscous solution (16 wt.% PVP aqueous solution), but very thick fibers with an average diameter of ~ 340 nm formed (Fig. 2c).

### Thixotropic behavior of TEMPO-CNF-added PVP aqueous solution

**Figure 3:**
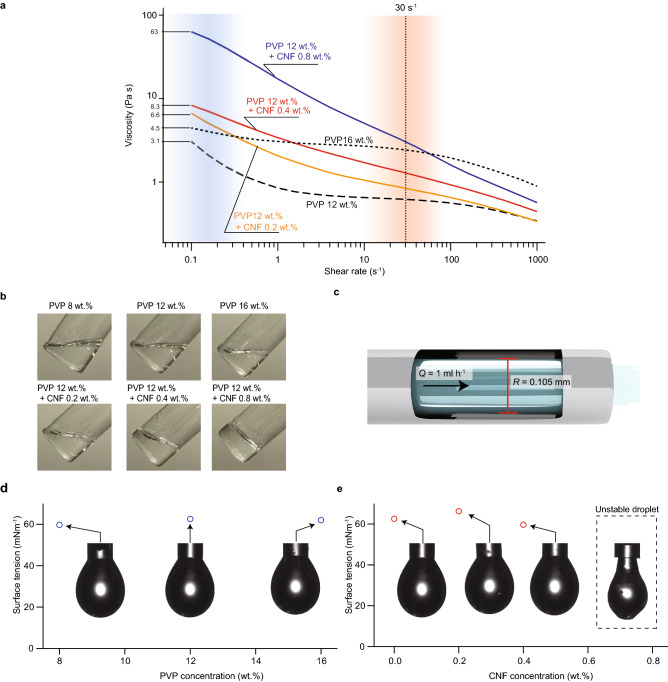
Rheological behavior of the prepared solutions for electrospinning. **a**, Relationships between viscosities and shear rates measured by a rheometer. **b**, Photos of prepared solutions captured directly after inclining by hand for approximately 1 s. While the PVP pristine solutions quickly respond to inclination, the TEMPO-CNF-added solutions exhibit a slower response (Supplementary Movie 1). **c**, Schematic image of the syringe needle, showing the inner diameter R and solution feed rate Q. Surface tension estimated from the shape of the droplet with respect to **d**, PVP concentration and **e**, TEMPO-CNF concentrations in the 12 wt.% PVP aqueous solution. The inset images in (**d**) and (**e**) depict the observed droplets. For the CNF concentration of 0.8 wt.%, the solution is too viscous to estimate the surface tension.

To observe the effect of adding TEMPO-CNF into the PVP aqueous solution on the fiber morphology, we prepared three more aqueous solutions containing 12 wt.% of PVP with 0.2, 0.4, and 0.8 wt.% of TEMPO-CNF. As expected, the viscosity increased with increasing TEMPO-CNF concentration and was 8 Pa·s even for the sample with only 0.4 wt.% TEMPO-CNF (Fig. 3a); the viscosity reached 63 Pa·s for 12 wt.% of PVP with 0.8 wt.% TEMPO-CNF. Figure 3b shows photos of solutions in a glass bottle after inclining for approximately 1 s; the TEMPO-CNF-added solutions exhibit a viscous nature. At this point, we prepared solutions with a sufficiently high viscosity that could overcome the surface tension of the nanofiber; however, such high viscosities (~ 10 Pa s) are out of the conventional range (typically below ~ 5 Pa·s) for electrospinning. Notably, in the DVT method, the high viscosity of the solution decreased significantly as the solution entered the syringe needle owing to the collapse of the network between the CNFs because of shear force (Fig. 1d). The shear force that the solution undergoes can be approximated by the following equation:

1 $$ \dot{\gamma } = \frac{4Q}{{\pi R^{3} }}. $$

where $\dot{\gamma }$ is the shear rate at the needle wall, *Q* is the volumetric flow rate, and *R* is the radius of the needle (Fig. 3c). The shear rate was estimated to be 30 s^−1^ for our experimental setup. The corresponding shear stress region is highlighted in Fig. 3a. At this region, PVP solutions containing TEMPO-CNF exhibited viscosity similar to those without TEMPO-CNF. This implies that the high viscosity attained by the addition of the TEMPO-CNF solution can be lowered inside the syringe needle owing to the thixotropy, and electrospinning should be possible. Figure 3d,e show the surface tension of the prepared solutions estimated by the pendant drop method. Error bars indicating standard deviation, which were below 1 mN m^−1^ for all the samples, are not shown in Fig. 3d, e because they are too small. A polymer solution is elongated when the electrical force overcomes the surface tension. Unlike the viscosity, we found that the surface tension of the TEMPO-CNF-added solutions was almost unchanged and was in a range similar to that of pristine PVP solutions in which electrospinning was successful. These findings confirm that the addition of TEMPO-CNF does not alter the surface tension, which is a force that needs to be overcome to prevent elongated fibers from breaking.

### Ultrafine fiber with TEMPO-CNF-added PVP aqueous solution

**Figure 4:**
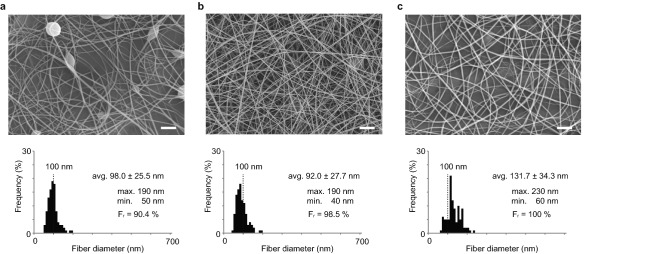
Effect of adding TEMPO-CNF into the 12 wt.% PVP aqueous solution. Electrospun fibers prepared by 12 wt.% PVP aqueous solution with (**a**) 0.2, (**b**) 0.4, and (**c**) 0.8 wt.% of TEMPO-CNF. Ultrafine nanofibers with an average diameter below 100 nm were achieved for 12 wt.% PVP aqueous solution with 0.4 wt.% TEMPO-CNF. Scale bars, 2 μm (**a**–**c**). Inset values are the average ± standard deviation. F_r_: fiber ratio.

Figure 4 shows SEM images of the electrospun samples prepared with different concentrations of TEMPO-CNF in 12 wt.% PVP aqueous solutions. For all three samples, we could obtain nanofibers by electrospinning. The low-concentration TEMPO-CNF solution (0.2 wt.%) achieved fibers with an average diameter of approximately 100 nm (Fig. 4a), which were thinner than those formed without TEMPO-CNF.

To determine the effect of TEMPO-CNF on the formation of the undesirable beads, we defined the fiber ratio (F_r_):

2 $$ {\text{F}}_{{\text{r}}} = \frac{{S_{fiber} }}{{S_{fiber} + S_{fiber} }} \times 100. $$

where S_*fiber*_ and *S*_*beads*_ are the traced fiber area and bead area, respectively (for further details, see Supplementary Note 1). F_r_ increased from 50% for a 12 wt.% PVP aqueous solution without TEMPO-CNF to ~ 90% with 0.2 wt.% TEMPO-CNF.

The number of beads further decreased with increasing TEMPO-CNF concentration to 0.4 wt.% (Fig. 4b), successfully demonstrating ultrafine, long, uniform fibers with an average diameter of ~ 90 nm and a considerably high F_r_ of 98.5%. For the highest-concentration sample (0.8 wt.% TEMPO-CNF), no beads were observed, but the fiber diameter increased to ~ 130 nm (Fig. 4c).

Solution conductivity is one of the important parameters affecting the fiber diameter and morphology; increase in conductivity decrease the fiber diameter, and thus NaCl is typically added to increase conductivity in electrospinning. To see how conductivity changes with the addition of small amount of TEMPO-CNF, we summarize the solution conductivity in Table 1 together with F_r_. We observed an increase in electric conductivity for the TEMPO-CNF-added solution. Specifically, the 12 wt.% PVP aqueous solutions without and with 0.4 wt.% TEMPO-CNF exhibited conductivities of 2 and 20.8 mS m^−1^, respectively, as presented in Table 1. To safely exclude the possibility that conductivity increase introduced by TEMPO-CNF significantly increased the fiber ratio, we investigated the morphology of electrospun fibers prepared using 12 wt.% PVP aqueous solution with 0.03 wt.% of NaCl, and we intended to see if conductivity alone could increase the fiber ratio without thixotropic behavior of the solution. Although the solution exhibited a conductivity of 47 mS m^−1^, which was more conductive than the 0.4 wt.% TEMPO-CNF-added 12 wt.% PVP aqueous solution and we see improvements in average fiber diameter and fiber ratio, the fiber ratio did not significantly increase to the degree that TEMPO-CNF containing solution exhibited (Supplementary Fig. 4), proving that the small charge introduced by CNF does not decrease the number of beads, and the increased fiber ratio was solely attributed to the viscosity recovery during electrospinning.

**Table 1:** Average diameter and estimated fiber ratio (F_r_) of prepared PVP electrospun textiles.

PVP concentration (wt.%)	NaCl concentration (wt.%)	TEMPO-CNF concentration (wt.%)	Average diameter (nm)	Fiber ratio, F_r_ (%)	Conductivity (mS m^−1^)
8	0	0	57.5	32.4	1.8
12	0	0	122.8	49.0	2.0
16	0	0	342.8	100	3.0
12	0	0.2	98.0	90.4	11.4
		0.4	92.0	98.5	20.8
		0.8	131.7	100	42.4
	0.03	0	103.0	64.7	47.0

## Discussion

In this work, we successfully electrospun a non-woven textile composed of uniform, ultrafine fibers, which has not been achieved thus far by simply adjusting the concentration of PVP or by adding salts to the electrospinning solution to add more charge. By adding TEMPO-CNF to the PVP aqueous solution, we made solution to be thixotropic and dynamically tuned the viscosity of the solution. While in a static state, a significantly high viscosity was obtained, but inside the syringe, this viscosity decreased back to an ordinary value at which electrospinning remained possible. We confirmed the thixotropic behavior of the solutions and ascribed it to the DVT stabilizing the produced elongated nanofiber before beads started to form after leaving the syringe needle and being freed from the large tensile stress owing to the surface charge, which repels the neighboring segments of the polymer in nanofiber and decreases the fiber diameter (Fig. 1d). To further verify this mechanism, we performed numerical analysis to track the time evolution of the fiber morphologies. The dynamics of a two-phase fluid consisting of PVP and water were simulated by solving the fluid dynamics equations with the aid of the volume fraction technique (see supporting information).

**Figure 5:**
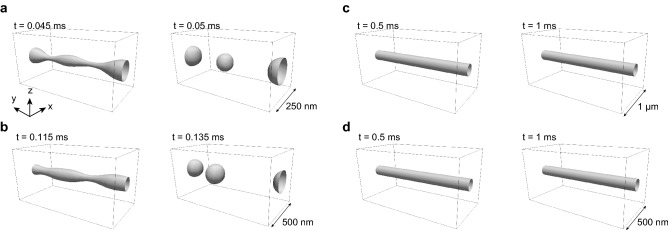
Simulated time-lapse changes in the fibers. Time evolution of fiber morphology up to 1 ms, starting with circular cylinders with diameters of (**a**) 50, (**b**) 100, and (**c**) 200 nm; the viscosity of the fiber material is 0.8 Pa·s. **d**, 100-nm-diameter fiber with a viscosity of 8 Pa·s (Supplementary Movie 2).

Figure 5a–c compares the typical simulated morphologies of PVP fibers having different diameters, obtained after time evolutions with the initial condition of a PVP circular cylinder soaked in water. Fibers with diameters of 50 and 100 nm exhibited fragmentation due to Rayleigh instability, which explains the instability of liquid jets, theorized a century ago^24^, finally breaking up into droplets in sub-milliseconds. The 200-nm-diameter fibers maintained their initial cylindrical shape for a much longer time, up to 1.4 ms, after which the same instability occurred. Importantly, this instability, which is driven by the high surface tension due to the high surface-to-volume ratio of the fine fibers, was shown to be significantly retarded simply by increasing the viscosity by one order of magnitude as shown in Fig. 5d. The increase in viscosity enabled us to maintain the original structure of a 100-nm-diameter fiber for a very long time (up to 4 ms), i.e., more than 30 times longer than for the original viscosity (0.8 Pa·s). We note here that the high viscosity, 8 Pa·s, is comparable to that viscosity realized by adding TEMPO-CNF, demonstrating the validity of our concept in experiments.

Finally, to discuss its versatility, we applied the DVT method to poly(acrylic acid) (PAA), another well-known water-soluble and biocompatible polymer other than PVP^25^. The addition of NaCl to PAA aqueous solution did not affect the rheological behavior of the solution (Supplementary Fig. 5), but it reduced the average fiber diameter from ~ 90 to ~ 80 nm due to the introduced surface charge or conductivity. There was no significant improvement in the fiber ratio and it was 55% for PAA aqueous solution containing 0.1 wt.% NaCl, as summarized in Supplementary Table 1. In contrast, upon adding TEMPO-CNF to this solution, viscosity increased similar to the PVP aqueous solution case (Supplementary Fig. 5); a significant improvement in fiber ratio (96%) was achieved and bead formation was successfully suppressed (Supplementary Figs. 6–7), demonstrating the versatility of this DVT method.

Accordingly, the concept of using the rheological behavior of the solution is distinct from previously proposed methods: while the conventional approach was intended to elongate the fiber as little as possible by adding salts, conductive fibers like carbon nanotubes, and other ingredients, the aim of using thixotropy was to stabilize the elongated fibers against Rayleigh instability. We believe that our approach provides a novel route to obtaining long, uniform ultrafine fiber textiles.

## Conclusions

In conclusion, we demonstrated a non-woven textile of nanofibers with a fiber diameter below 100 nm by DVT, and we successfully suppressed the formation of beady structures by adding TEMPO-oxidized cellulose into the electrospinning solution.

Our DVT method relies on the high thixotropy of the solution induced by adding small amount of TEMPO-CNF, which exhibited high viscosity in a static state but was less viscous during electrospinning. After discharge from the syringe needle and being freed from tensile stress during electrospinning due to the repulsion of charged segments of nanofiber, the viscosity was restored to a high value (Fig. 1d), successfully overcoming the Rayleigh instability and preventing the transition of the ultrafine nanofiber into beads. Our numerical simulations validated that the viscosity of the solution is a key parameter enabling this stabilization. We also confirmed that the small charge introduced by TEMPO-CNF did not decrease the number of beads, and the increased fiber ratio was solely attributed to the viscosity recovery during electrospinning. We applied DVT to aqueous biocompatible polymer (PVP and PAA) solutions to achieve a bead-free nanofiber textile; however, considering the working principle of DVT in electrospinning, similar effects are expected using other rheology agents, e.g. hydrophobic fumed silica^26,27^, and for a variety of polymers that are only soluble in non-aqueous solutions. Further study in this direction will be conducted in the future.

Unlike previous efforts to obtain nanofibers using additives to tune the conductivity and surface tension of the electrospinning solution to elongate the polymer, we adopted a completely different concept of exploiting the thixotropy of the solution to stabilize the elongated nanofiber. Fine fibers are important for improving the performance of textiles in various applications. This approach further improves upon on a well-established electrospinning technique, and these results pave the way for preparing ultrafine nanofiber textiles with a high surface made of non-toxic, biocompatible polymers for a wide variety of applications.

## Methods

### Materials

PVP (Mv ~ 1,300,000), PAA (Mv ~ 450,000) and NaCl was acquired from Sigma Aldrich, and TEMPO-CNF (Rheocrysta) from Daiichi kogyo. The PVP, H_2_O (18.2 MΩ), and TEMPO-CNF were weighed to prepare the following six samples: 8, 12, and 16 wt.% aqueous solutions of PVP, and mixed solutions containing 12 wt.% aqueous solutions of PVP with 0.2, 0.4, and 0.8 wt.% TEMPO-CNF. The PAA and H_2_O were weighed to prepare the 3 wt.% aqueous solution of PAA. 0.03 to 0.11 wt.% of NaCl was added to this 3 wt.% aqueous solution of PAA to modify the solution electrical conductivity.

### Electrospinning

We performed electrospinning using a single disposable syringe (6 mL, 27G syringe) and a stainless steel collector. A potential (15 kV) was applied to the PVP solution, and a potential of 7.5 kV was applied for the PAA solutions between the syringe and collector. The solution feed rate was fixed at 1 mL h^−1^, and the distance between the syringe and collector was fixed at 15 cm. The duration of the electrospinning was set to be ~ 5 min. All the electrospinning was performed at 25 °C and humidity of 30–50% in air. The textiles were reproducible under these controlled conditions.

### Characterization

The conductivities of the prepared aqueous solutions were measured using a conductivity meter (HORIBA Co. Ltd., LAQUA (F-74) with conductivity measurement cell (3,552-10D)) at 25 °C.

The surface tension was estimated using the standard pendant drop method, wherein a droplet (15 μL) formed using a 20 G needle was photographed for 10 s after the formation of the droplet using a contact angle analyzer (B100W, Asumi-giken) followed by analysis of the image using the Young–Laplace equation ^28^. The measurement was repeated five times. Before the measurement, the syringe and needle were washed thoroughly using isopropyl alcohol followed by repetitive filling and expelling of the sample solution from the needle (Supplementary Fig. 8).

The viscosity or rheological behavior of the PVP solutions was evaluated using a rotating rheometer (TA Instruments, ARES G2). The viscosities of the solutions were measured at shear rates between 10^−1^ and 10^3^ s^−1^. The electrospun samples were characterized by SEM (HITACHI High-Technologies, S3600N and S-5500). Au was deposited less than 4 nm estimated by quartz thickness monitor on the prepared electrospun samples with a desktop magnetron sputtering system (HITACHI High-Technologies, MC1000) to obtain clear SEM images. A total of ~ 100 fibers were randomly selected from the SEM images for calculating the average diameter and standard deviation.

### Simulation

The break-up of fibers was simulated using the open-source code OpenFoam^29^. The interFoam solver for multiphase analysis was employed; herein, the two phases represented PVP and water, Specifically, the values of the density and the viscosity of water were 1 g cm^−1^ and 0.001 Pa·s, whereas those of PVP were 1.2 g cm^−1^ and 0.8 Pa·s (pure PVP) or 8 Pa·s (TEMPO-CNF-added PVP); the surface tension of the interface was assumed to be 70 mN m^−1^. Here, we considered fibers having diameters D = 50, 100, and 200 nm. The size of the simulation box was 5D × 10D × 5D, where the *y* axis was in the longitudinal direction of the fiber. The periodic boundary condition was assumed in the *x* and *y* directions, whereas the non-slip wall was placed at the bottom in the *z* direction, and the constant-pressure boundary condition was applied at the top in the *z* direction. The grid interval was D/16 in the *x* and *z* directions and D/8 in the *y* direction.

## Supplementary information

Supplementary file 1.

Supplementary file 2.

Supplementary file 3.
