# Supplementary material for: Dynamic viscosity recovery of electrospinning solution for stabilizing elongated ultrafine polymer nanofiber by TEMPO-CNF
Source: Sci Rep. 2020 Aug 10;10:13427. doi: 10.1038/s41598-020-69136-2 (PMC7417572; doi:10.1038/s41598-020-69136-2)
Supplement: Supplementary file 1 [file 41598_2020_69136_MOESM1_ESM.docx]

Supporting Information for

Dynamic viscosity recovery of electrospinning solution for stabilizing elongated ultrafine polymer nanofiber by TEMPO-CNF

Shougo Higashi*, Takayuki Hirai, Masato Matsubara, Hiroaki Yoshida, and Atsushi Beniya

Correspondence to Shougo Higashi: [shigashi@mosk.tytlabs.co.jp](mailto:shigashi@mosk.tytlabs.co.jp)

Supplementary Note 1 | Determining the fiber ratio

To estimate the fiber ratio (F_r_), the region containing only fibers in the SEM image was traced manually using Photoshop Elements (Adobe Co. Ltd., ver. 8.0). We also traced the bead region to estimate the number of beads. To avoid underestimating the number of beads, we manually outlined the beads hidden behind other beads, and we measured the traced fiber area (S*_fiber_*) and bead area (*S_beads_*) using ImageJ^1, 2^ and estimated the fiber-to-bead based on Equation (2) in the main text.

Supplementary Movie 1 | Dynamic behavior of the prepared solutions

This movie illustrates the dynamic behavior of the prepared solutions after inclining by hand. The upper panels show the pristine aqueous PVP solutions of concentrations 8, 12, and 16 wt.%. The lower panels show 12 wt.% PVP solution with CNF added in concentrations of 0.2, 0.4, and 0.8 wt.%.

Supplementary Movie 2 | Time evolution of the fiber morphology

This movie shows the time evolution of the fiber morphology until the fibers break up and form drops, starting with circular cylinders with diameters of 50, 100, and 200 nm; the viscosity of the fiber material is 0.8 Pa s, except for that in the right-bottom panel, in which the diameter is 100 nm and the viscosity is 8 Pa s.

**Supplementary** **Table 1. Average diameter and estimated fiber ratio (Fr) of the electrospun PAA fabrics.**

| PAA concentration wt.% | NaCl wt.% | TEMPO-CNF concentration wt.% | Average diameter  (nm) | Fiber ratio (%) | Conductivity (mSm^-1^) |
| --- | --- | --- | --- | --- | --- |
| **3** | **0** | **0** | **88.3** | **51.2** | **105.6** |
|  | **0.05** | **0** | **84.4** | **64.1** | **231.0** |
|  | **0.1** | **0** | **82.2** | **54.9** | **292.0** |
|  | **0.11** | **0** | **78.4** | **55.0** | **336.0** |
|  | **0.1** | **1.2** | **77.7** | **96.0** | **316.0** |


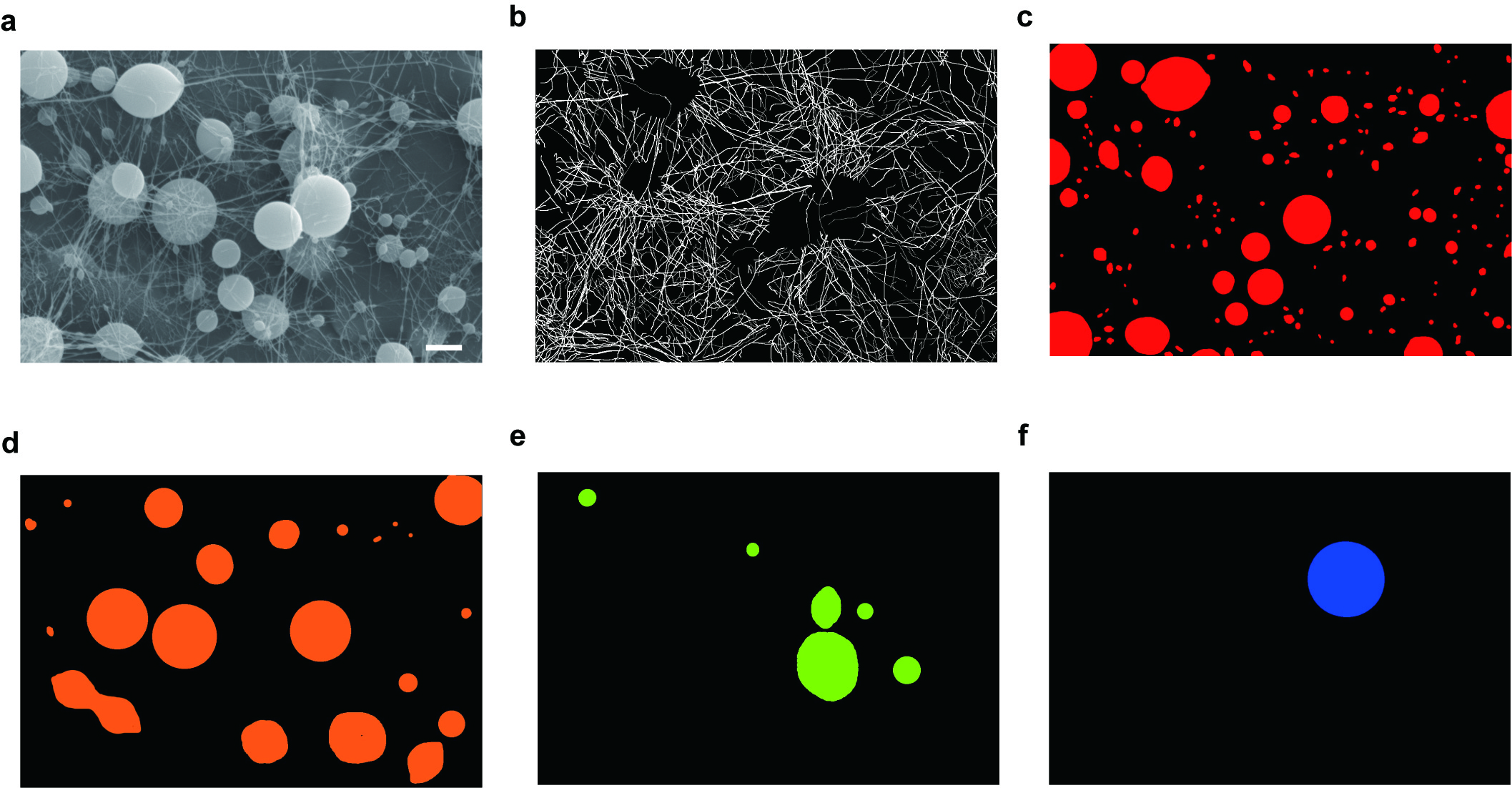


**Supplementary** **Fig. 1** | Electrospun PVP nanofibers prepared using 8 wt.% PVP aqueous solution. **a**, SEM image of electrospun PVP fibers with beads prepared using 8 wt.% PVP aqueous solution. **b**, Processed SEM image that identifies the fibers and beads (**c**-**f**). Some beads overlap others in the image. To avoid underestimating the number of beads, we manually identified the beads behind other beads. Scale bar in (**a**), 2 μm.


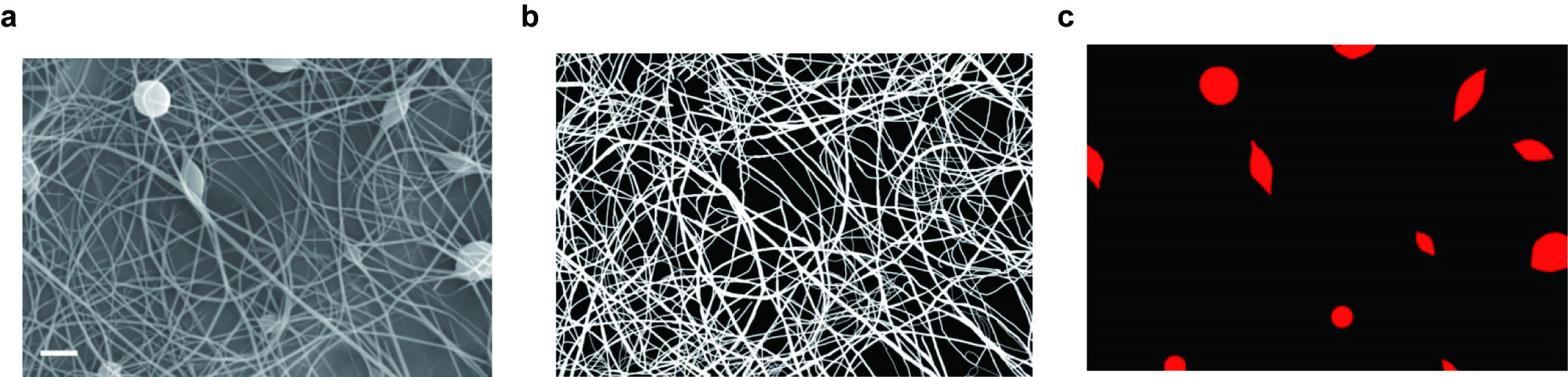


**Supplementary** **Fig. 2** | Electrospun PVP nanofibers prepared using 12 wt.% PVP aqueous solution with 0.2 wt.% TEMPO-CNF. **a**, SEM image of electrospun PVP fibers with beads prepared using 12 wt.% PVP aqueous solution with 0.2 wt.% TEMPO-CNF. Processed SEM image that identifies the fibers (**b**) and beads (**c**). Scale bar in (**a**), 2 μm.


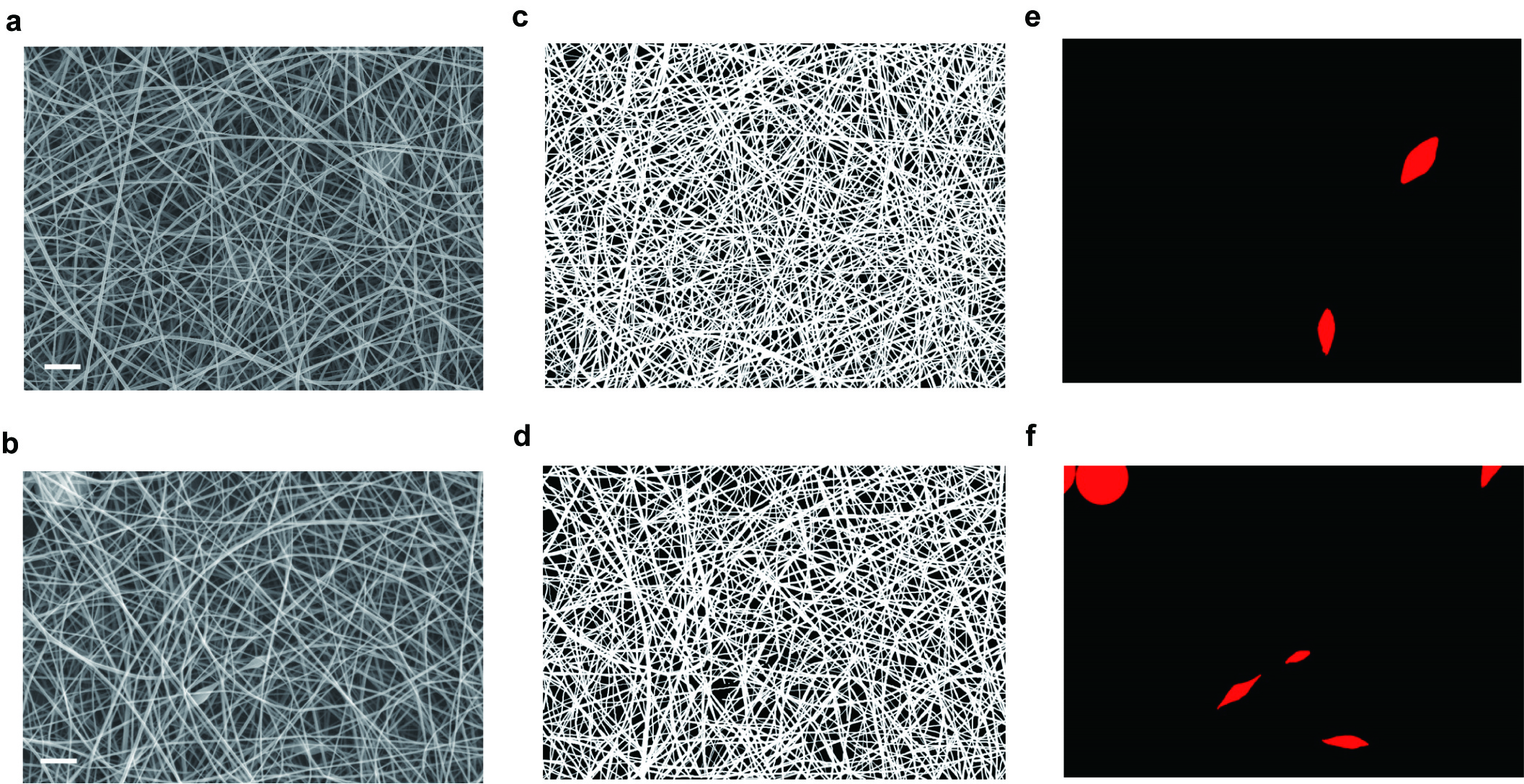


**Supplementary** **Fig. 3** | **Electrospun PVP nanofibers prepared using 12 wt.% PVP aqueous solution with 0.4 wt.% TEMPO-CNF. a**,**b**, SEM image of electrospun PVP fibers with beads prepared using 12 wt.% PVP aqueous solution with 0.4 wt.% TEMPO-CNF. (**c**,**d**) and (**e**,**f**) are the processed SEM images that identify the fibers and beads, respectively. Note that the two different SEM images (**a**,**b**) were acquired from the same sample. Scale bars, 2 μm (**a**.**b**)


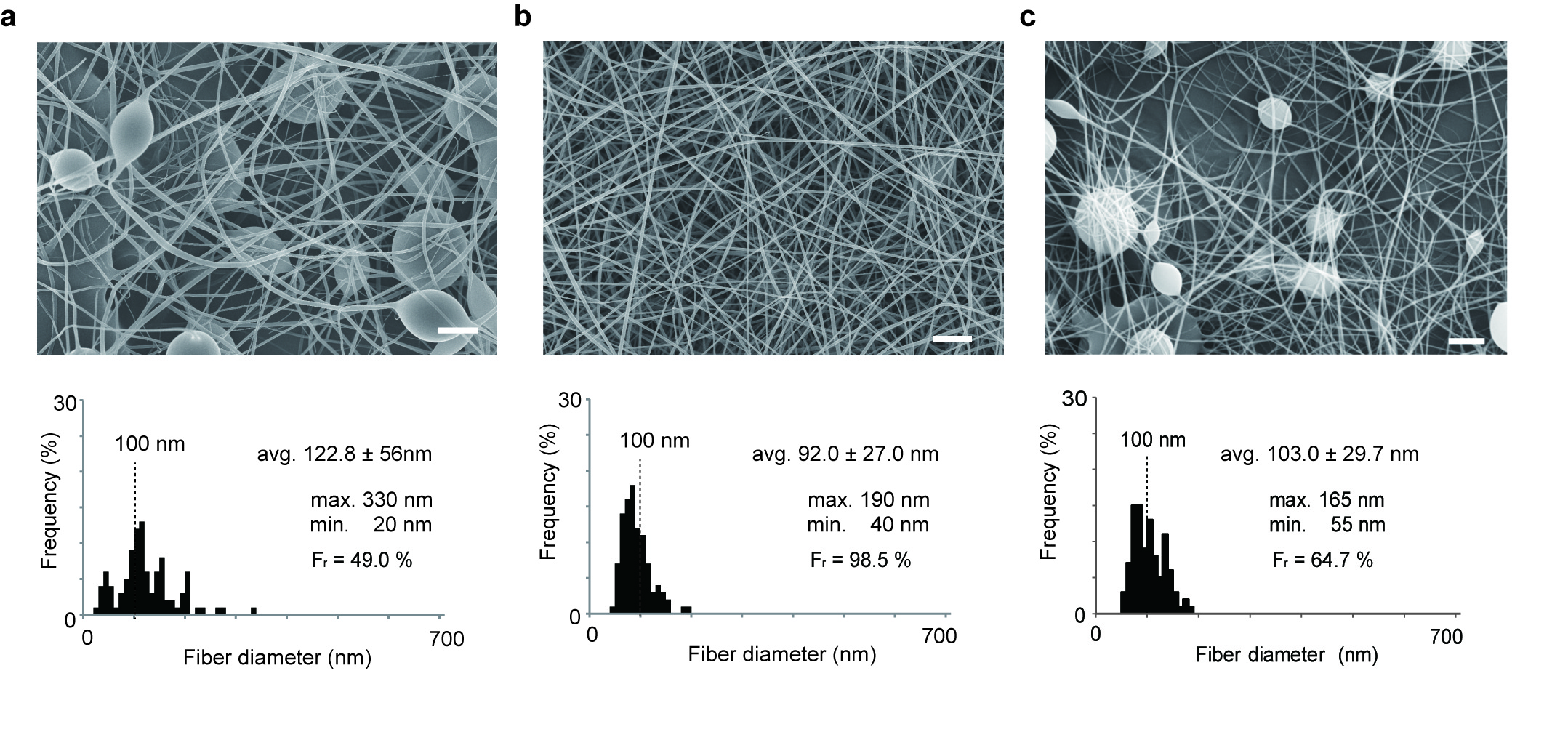


**Supplementary** **Fig. 4** | **Comparison of fiber morphology of TEMPO-CNF-added 12 wt. % PVP aqueous solutions with and without 0.03 wt.% NaCl.**

SEM images of fibers from the 12 wt.% PVP aqueous solution (**a**) without and (**b**) with 0.4 wt.% CNF. The formation of beads was successfully suppressed. The addition of 0.4 wt.% CNF increased the conductivity from 2 to 20 mS m^-1^. **c**, 12 wt.% PVP aqueous solution with 0.03 wt.% NaCl, which exhibits a conductivity of 47 mS m^-1^ (Table 1). If the charge introduced by the CNF is the reason for the suppression of the bead formation, the solution having the same charge should also show the same suppression effect. However, we did not observe any such improvements, which implies that the key factor in increasing the fiber-to-bead is neither the charge nor the surface tension but indeed the viscosity. The scale bars represent 2 μm.


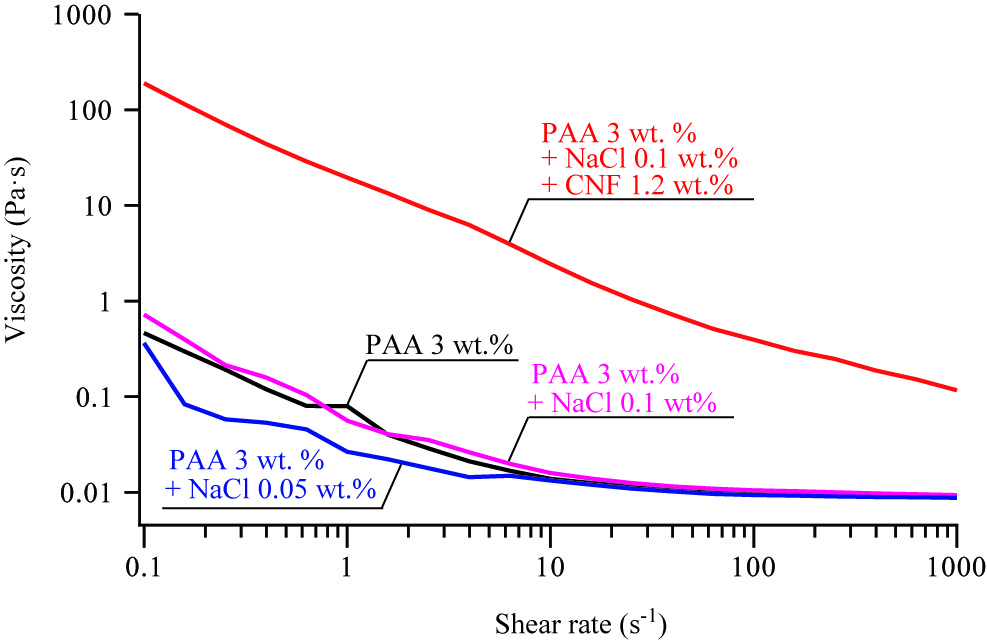


**Supplementary** **Fig. 5 |** **Rheological behavior of PAA solutions for electrospinning.**

3 wt.% PAA aqueous solution with different NaCl concentrations and 3 wt.% PAA with both of NaCl (0.01 wt.%) and CNF (12 wt.%) are compared. We confirmed that NaCl addition does not increase the viscosity of the PAA aqueous solution.


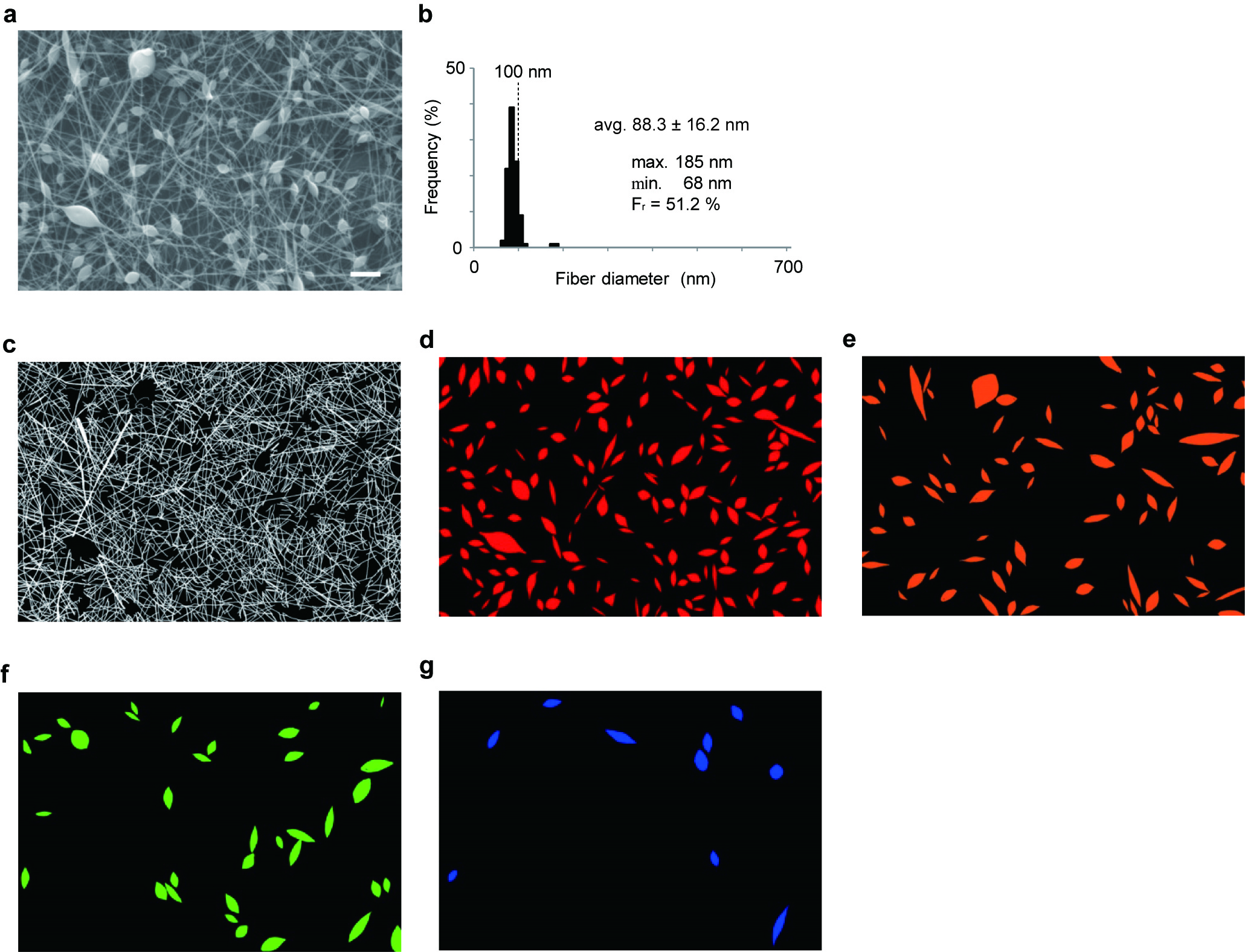


**Supplementary** **Fig. 6 |** **Electrospun fibers using 3 wt.% PAA aqueous solution. a**, SEM image of electrospun PAA fibers with beads prepared using 3 wt.% PAA aqueous solution. **b**, size distribution of electrospun fibers in the SEM image. **c**-**g**, Processed SEM images that identify the fibers (**c**) and beads (**d**-**g**).


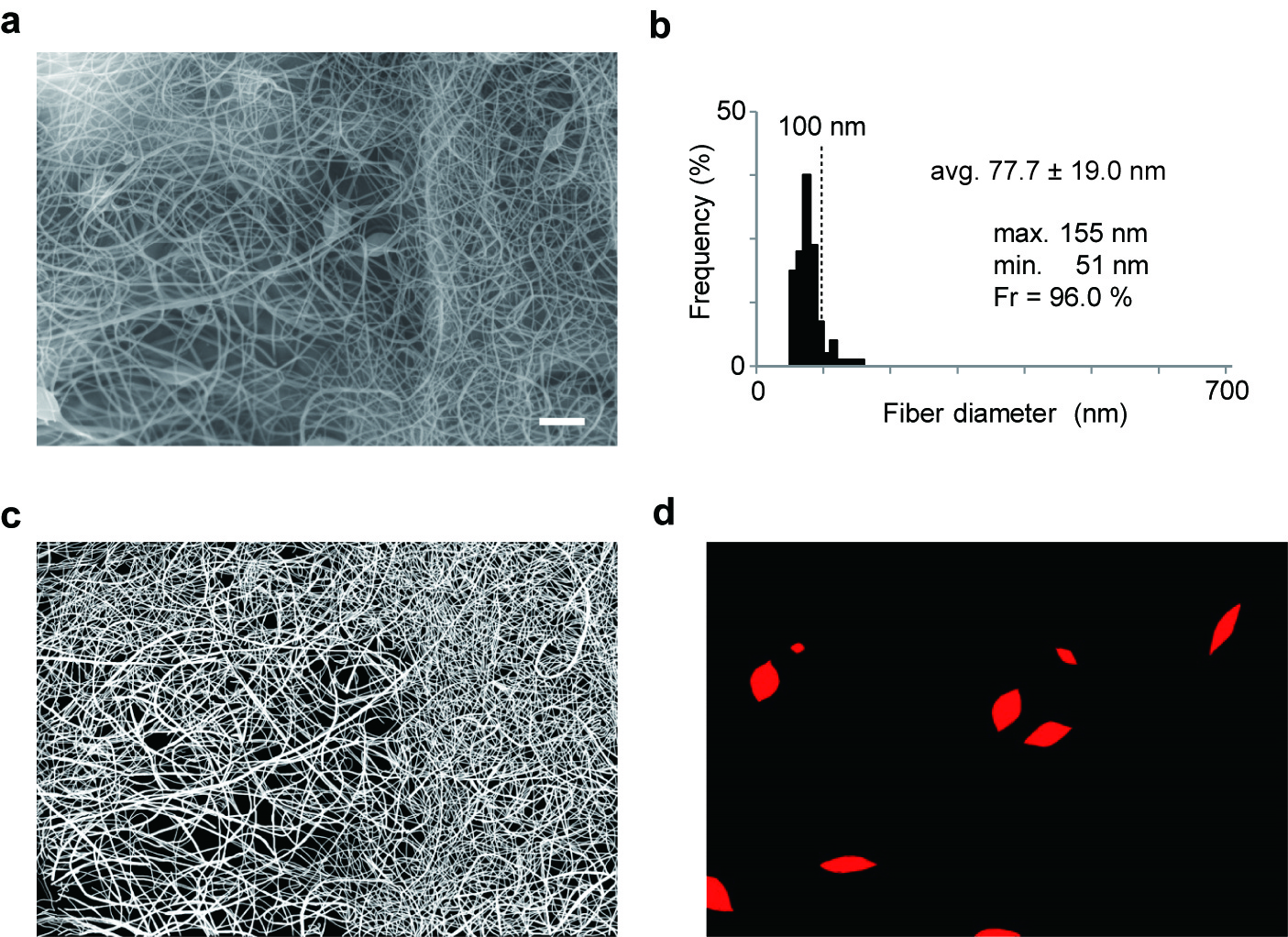


**Supplementary** **Fig. 7 |** **Electrospun fibers using 3 wt.% PAA aqueous solution containing both 1.2 wt.% TEMPO-CNF and 0.1 wt.% NaCl. a**, SEM image of electrospun PAA fibers prepared using 3 wt.% PAA aqueous solution with both 1.2 wt.% TEMPO-CNF and 0.1 wt.% NaCl. **b**, Size distribution of electrospun fibers in SEM image. **c**, Processed SEM image that identifies the fibers and beads(**d**).


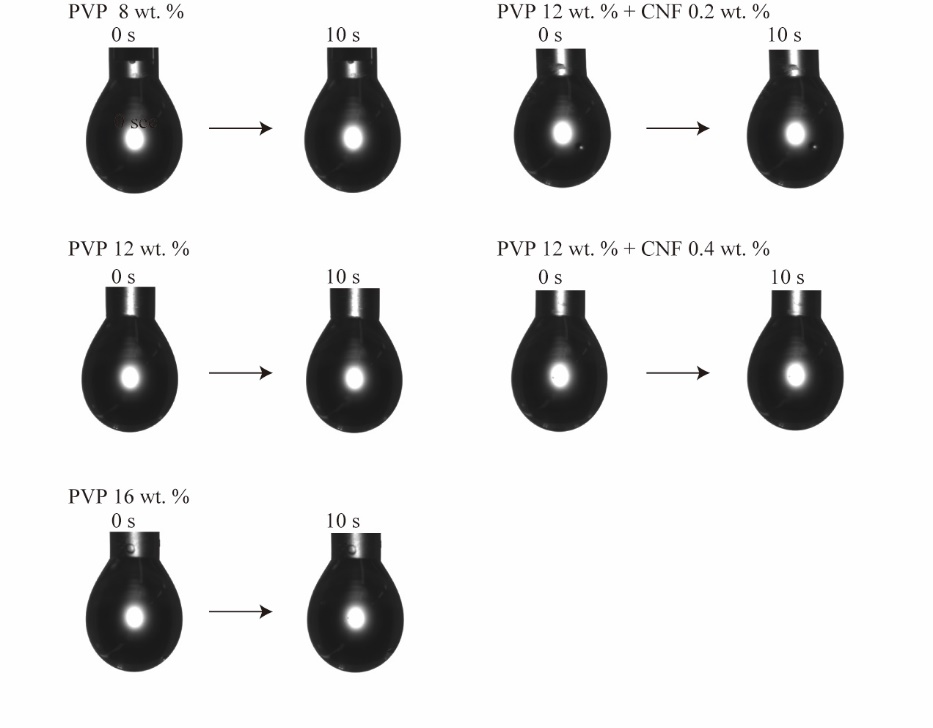


**Supplementary** **Fig. 8** | **Chronological change of the 15-μL droplets.** Images of the droplets formed immediately after the formation of the 15-μL droplets (denoted as 0 s) and those formed 10 s later. There was no significant difference between the droplet images obtained at 0 s and 10 s. These observations ensured that the droplets were measured at the equilibrium state.

**Supplementary References**

1. Rasband, W.S., ImageJ, U. S. National Institutes of Health, Bethesda, Maryland, USA, <http://imagej.nih.gov/ij/>, 1997-2012

2. Schneider, C.A., Rasband, W.S. & Eliceiri, K.W. NIH Image to ImageJ: 25 years of image analysis. *Nature Methods* **9**, 671-675 (2012).
